# Supplementary material for: Large magnon-induced anomalous Nernst conductivity in single-crystal MnBi
Source: Joule. 2021 Nov 17;5(11):3057–67. doi: 10.1016/j.joule.2021.08.007 (PMC8604385; doi:10.1016/j.joule.2021.08.007)
Supplement: Document S1. Supplemental experimental procedures, Figures S1–S8, and Table S1 [file mmc1.pdf]

Joule, Volume *5*

Supplemental information

Large magnon-induced anomalous

Nernst conductivity in single-crystal MnBi

Bin He, Cüneyt Şahin, Stephen R. Boona, Brian C. Sales, Yu Pan, Claudia Felser, Michael E. Flatté, and Joseph P. Heremans

Supplemental Experimental Procedures

1 Single crystal characterization

High Quality single crystals are essential to study the thermoelectric transport properties of materials. In this study, the single crystal information of Batch 1 can be found from previous publication (Ref. 26 in the main text). In this part we show the crystal information of Batch-2 grown at the Max Planck Institute. Fig. S1(a) shows the optical image of MnBi single crystal from Batch-2, with a hexagon shape. Fig. S1(b) shows the Laue diffraction taken on the crystal. According to the Mn-Bi binary phase diagram, MnBi single crystals can be grown via flux method with a starting Mn mole fraction less than 10%. As described in the main text, Batch 1 and 2 were grown with 6% and 9% Mn respectively. By analyzing the ordinary Hall effect, they have carrier concentrations of ~ $8 \times 10^{20}/cm^3$ and $1.4 \times 10^{21}/cm^3$. We suspect the crystals are with different defect structures, which give rise to the different carrier concentrations. The defect structure is affected by the Mn chemical potential in the crystal growth process. To our knowledge, we believe a lower starting Mn composition is beneficial for low carrier concentration and larger thermoelectric/thermomagnetic response.

(a) (b)

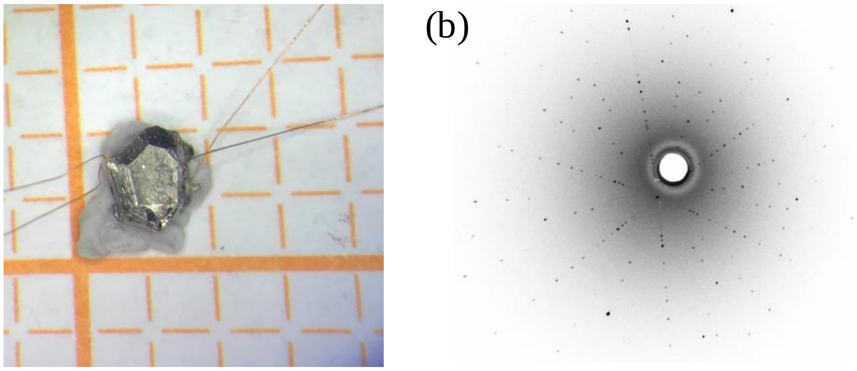

Figure S1: Characterization of single crystal MnBi. (a) Optical image and (b) Laue diffraction pattern on MnBi single crystal from Batch-2, indicative of high crystal quality

	Starting composition	Carrier density	Maximum S_{ANE}	Maximum α_{ANE}
Batch-1	6% Mn+94% Bi	$8 \times 10^{20}/cm^3$	10 μV/K	44A/Km
Batch-2	9% Mn+91%Bi	$1.4 \times 10^{21}/cm^3$	7 μV/K	~28A/Km

Table S1: Comparison between the crystals Batch-1 and Batch-2. With a higher starting Mn concentration, the carrier concentration of Batch-2 is higher than that of Batch-1, resulting in a smaller magnon-drag induced ANE thermopower and ANE conductivity.

Since batch 2 has a higher carrier concentration than batch 1, which then results in smaller magnon-drag transport signals. In the magnon-drag picture for longitudinal thermopower, the magnon-drag thermopower can be expressed as $S_{md} = \frac{2}{3}\frac{C_m}{ne}\frac{1}{1+\frac{\tau_{em}}{\tau_m}}$, where C_m is the magnon heat capacity, n is the carrier concentration, e is elemental charge, τ_{em} is the electron magnon scattering time and τ_m is the magnon mean free time. Generally, the magnon-drag thermopower is inversely proportional to the carrier concentration. The magnon-drag ANE may not have such simple relation. But it can be inferred that the magnon-drag ANE should be negatively related to the carrier concentration, which explains the sample to sample variation.

2 Magnetic Properties of MnBi

Fig. S2 (a) and (b) show the magnetization vs. field (M-H) at various temperatures when the applied field is parallel to *c*-axis and *ab*-plane, respectively. Our measurements mainly focus on the SR temperature range of 80 K and above, consistent with our transport measurement. Above 90 K, when the external field is applied parallel to *c*-axis, the spins can be easily aligned parallel to field, with the saturation field increasing upon cooling. The opposite trend has been observed when the field is applied in *ab*-plane. The crystal reaches its saturation magnetization at 80 K, with a small saturation field of 0.6 T. When the temperature is higher than 90 K, the saturation field scales with temperature, with the *c*-axis becoming the easy axis above 140 K. The saturation field is greater than 2 T at 180 K, which is beyond the max field we applied to our crystals. We also notice that, by applying high field, the crystals break into pieces after taken out from the MPMS. Thus, we do not apply field higher than 1.5 T for our transport measurement.

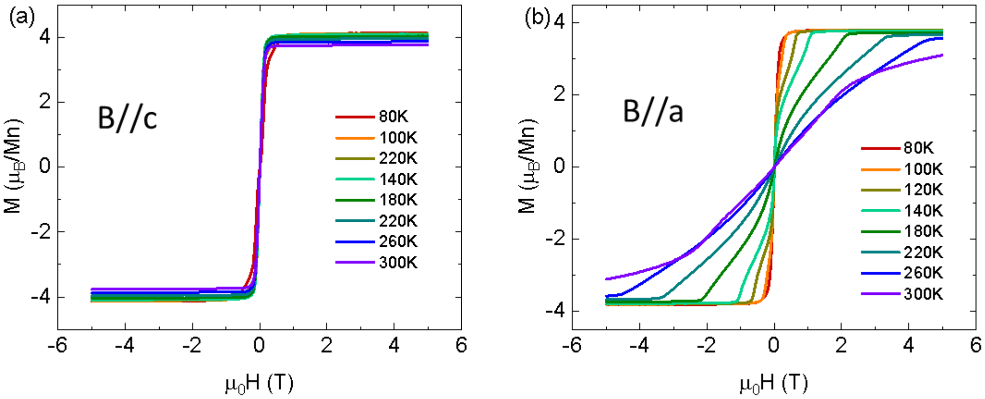

Figure S2: M-H results of MnBi single crystals. (a) M-H curve with external field along [0001]. (b) M-H curves with external field along $[2\bar{1}\bar{1}0]$. Data are taken on two small crystals from Batch-2. A clear spin-reorientation process is observed from M-H measurement.

3.Sample oxidation

The reproducibility of the transport data is established in the main text on freshly-grown samples. However, due to sample oxidation problems, it must be noted that the thermopower data change with the thermal cycling of the samples, and the Nernst data do so too although to a lesser extent. Compared to other manganese pnictides, MnBi single crystals have limited air stability because the electronegativity of Bi is small compared to other anions. We observed that after each thermal cycling, the surface of the MnBi crystals turned slightly yellow, indicative of the formation of bismuth oxide or manganese oxides. Thus, it is highly important to guarantee that the sample is mounted and measured in an air-free atmosphere to prevent sample oxidation and the accompanied weakening of the anomalous Nernst effect (ANE).

Here, we show the thermoelectric transport properties of sample $B1_{\perp}$ over three thermal cycles. We measured the sample multiple times for reproducibility; thus, we were able to observe a clear trend that both the ANE and thermopower decrease, which can come from oxidation of the sample. When MnBi crystals are oxidized, both the Mn and Bi are able to grab oxygen from the air, forming bismuth oxide Bi_2O_3, and complex manganese oxides (like MnO_2). This oxidation is creating defects and damaging sample quality. Oxidation of the crystal introduces a secondary phase and changes the carrier concentration of the sample, which can significantly affect the transport properties of the single crystal. Fig. S3(a) shows the anomalous Nernst thermopower of sample $B1_{\perp}$ at 100 K. The pristine sample had even larger anomalous Nernst thermopower in the first measurement. As we thermally cycled the sample in the cryostat, the anomalous Nernst thermopower reduced to 10 μV/K for the second thermal cycle. After we remounted the sample for the PPMS measurement, the anomalous Nernst thermopower further reduced to about 4 μV/K. As discussed above, this reduction in the ANE comes from the oxidation of the sample. The induced oxide impurities significantly scatter the electrons and reduce the mean free path of them. As the magnon electron interaction is a dynamical process, the extra scattering brings the system back to equilibrium, leading to a smaller anomalous Nernst thermopower. Fig. S3(b) shows the thermopower of sample $B1_{\perp}$ over three cycles. We observed a decrease in thermopower as we conducted the measurements, which is consistent with the vanishing ANE. The increasing density of defects deflects the magnon

electron interaction, which further reduces the magnon-drag thermopower. For the third run, the sample shows a metallic behavior, resulting from the disappearance of magnon-drag contribution.

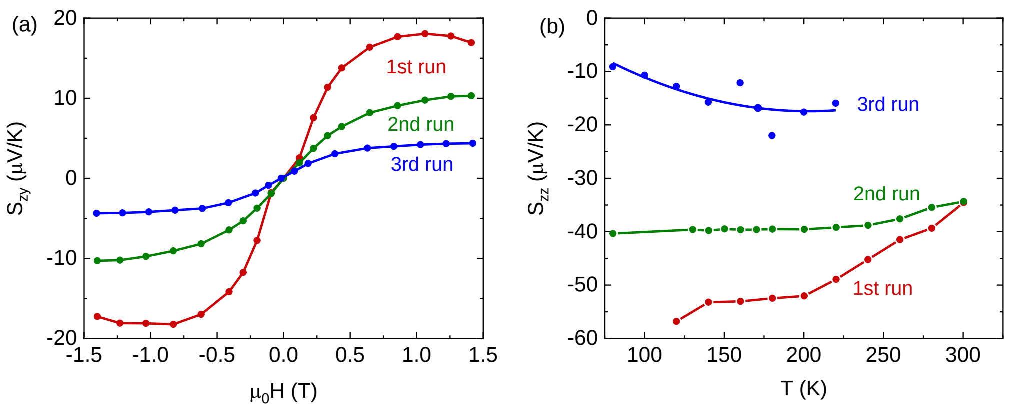

Figure S3: Thermal cycling results of thermoelectric transport coefficients. (a) Nernst thermopower of sample B1⊥ at 100 K after each thermal cycling. The anomalous Nernst thermopower decreases monotonically over thermal cycles. (b) Seebeck coefficient of B1⊥ after thermocycling. The absolute value of the Seebeck coefficient and anomalous Nernst coefficient decrease as the sample is oxidized and more defects are generated, possibly due to the vanishing of magnon-drag contribution.

4. Correlation between thermal and thermomagnetic transport properties

To further illustrate the possible contributions of magnons to the transport properties, we present the two samples with distinct thermal transport properties. We name our samples as B1⊥ and B1⊥*, where B1⊥ is the same sample as in the main text and B1⊥* is a sample which is unintentionally exposed to air for some time and thus got oxidized. As described in part S3, oxidation with damage the sample quality and reduce the ANE signal. Fig. S4(a) and S4(b) compare the ANE signals of the two samples. At 80 K, the ANE of B1⊥* is only 4 μV/K while the ANE of B1⊥ reaches 10 μV/K. As the temperature goes up, the ANE of B1⊥* increases with temperature as expected for intrinsic contribution, while the ANE of B1⊥ is almost constant over the spin reorientation process. At 160 K, the ANE of the two samples are almost identical to each other. The further decrease of B1⊥ is mainly due to non-saturation magnetization. However, since MnBi has strong magnetic anisotropy, we are unable to measured higher field results, as B1⊥* was teared apart by the applied field at 180 K. Moreover, the thermopower is also reduced as shown in Fig. S4(c). After being oxidized, the cross-plane thermopower shows a classic metallic signature, which is

increasing with temperature, with the absolute value much smaller than the pristine sample. Both of these two phenomena can be explained as the absence of magnon contribution. When the sample got oxidized, the magnon induced transport are suppressed, due to the higher defect density and smaller magnon and electron mean free path. At last, Fig. S4(d) shows the thermal conductivities of two samples $B1_{\perp}$ and $B1_{\perp}^{*}$. After oxidation, the thermal conductivity is significantly reduced at low temperature, due to the much smaller contribution of magnon thermal conductivity.

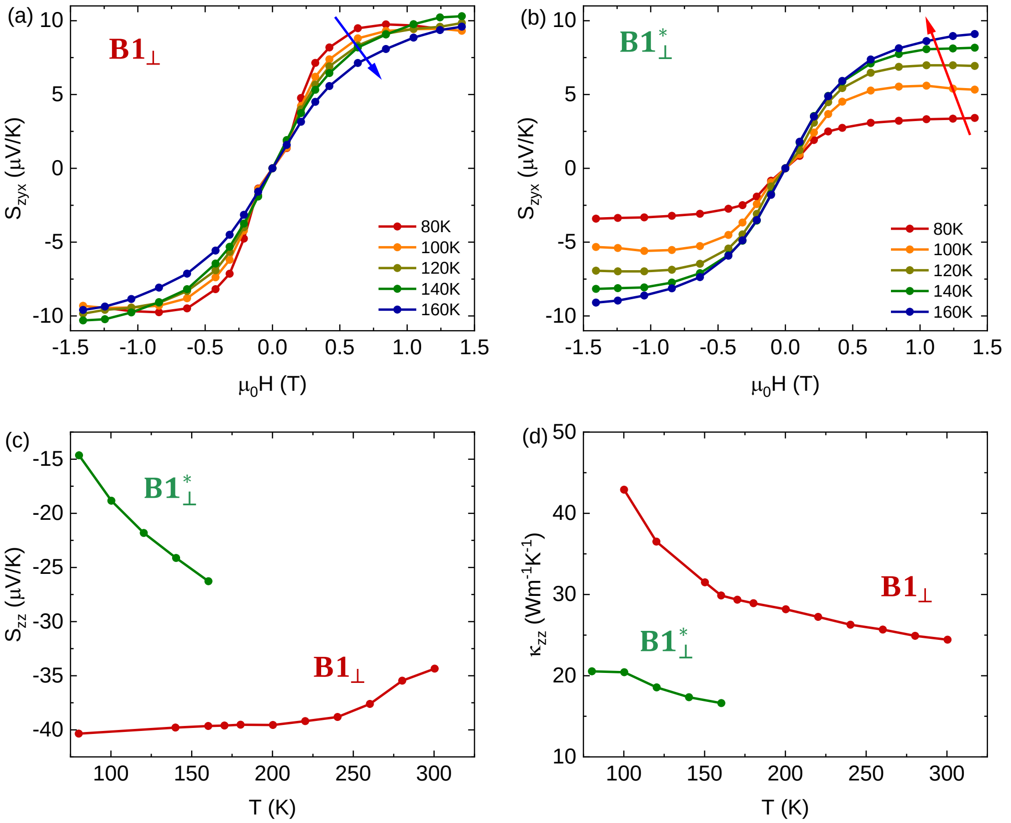

Figure S4: Relation between the large ANE thermopower and longitudinal Seebeck coefficient. (a) and (b) Nernst thermopower of sample $B1_{\perp}$ and $B1_{\perp}^{*}$ with opposite temperature dependence due to the unintentional oxidation of sample $B1_{\perp}^{*}$. (c) and (d) Seebeck coefficient and thermal conductivity comparison of samples $B1_{\perp}$ and $B1_{\perp}^{*}$. We clearly see the large Nernst signal is accompanied with a larger Seebeck coefficient and higher thermal conductivity.

5. Tight-binding calculation with a variation of Fermi energy

In the tight -binding calculation, it is essential to locate the position of the Fermi energy, in order to calculate the transverse thermoelectric conductivity α_{zyx} and α_{xyz} . We

first calculate the cross-plane anomalous Hall conductivity (AHC) as a function of Fermi energy, shown in Fig. S4(a). By comparing the experimental result (800 S/cm) to the theoretical value, we locate our Fermi energy at the position of 0 eV. With this Fermi level position, the temperature dependent α_{zyx} and α_{xyz} and then calculated and shown in the main text Fig. 4(c). Furthermore, to eliminate the unintentional error from any slight variation of the Fermi energy, we calculate the energy dependent α_{zyx} from -5 eV to 5 eV. The maximum α_{zyx} is always below 1A/Km, which can not explain the experimental value.

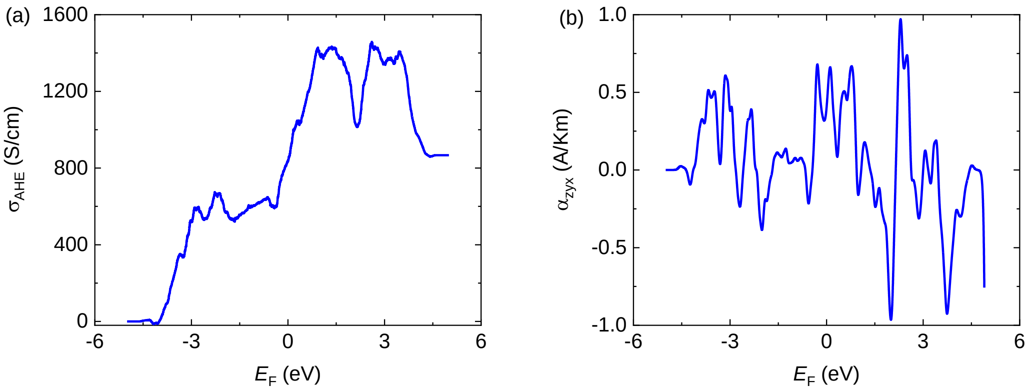

Figure S5: Energy dependent anomalous Hall and Nernst conductivities. (a) Cross-plane anomalous Hall conductivity (AHC) as a function of Fermi energy. Particularly when E_F=0 eV, the AHE is ~ 800 S/cm, which agrees with the experimental results. The Fermi energy is located at 0 eV for the transverse thermoelectric conductivity calculation. (b) Cross-plane transverse thermoelectric response element α in the tight-binding calculation, with the variation of the Fermi energy from -5 eV to 5 eV. In such a wide range of calculation, the calculated α is between -1 A/Km to 1 A/Km, indicative of the stability of the intrinsic contribution of the transverse thermoelectric response. Meanwhile, we conclude that, our giant transverse thermoelectric response cannot be explained by simply adjusting the position of the Fermi energy.

6. Complete dataset of the Nernst signals

In this part we present the Nernst signals before the subtraction of the anomalous part. Fig. S6 (a) – (d) show the total Nernst thermopower after anti-symmetrization from the raw data. We observe a clear slope change after saturation magnetization, as a result of ordinary Nernst effect (ONE). Meanwhile, as temperature goes up, we find a sign change of the ONE part. As the ONE is related to the scattering mechanism, there should be a change in the scattering mechanism before and after the spin reorientation effect.

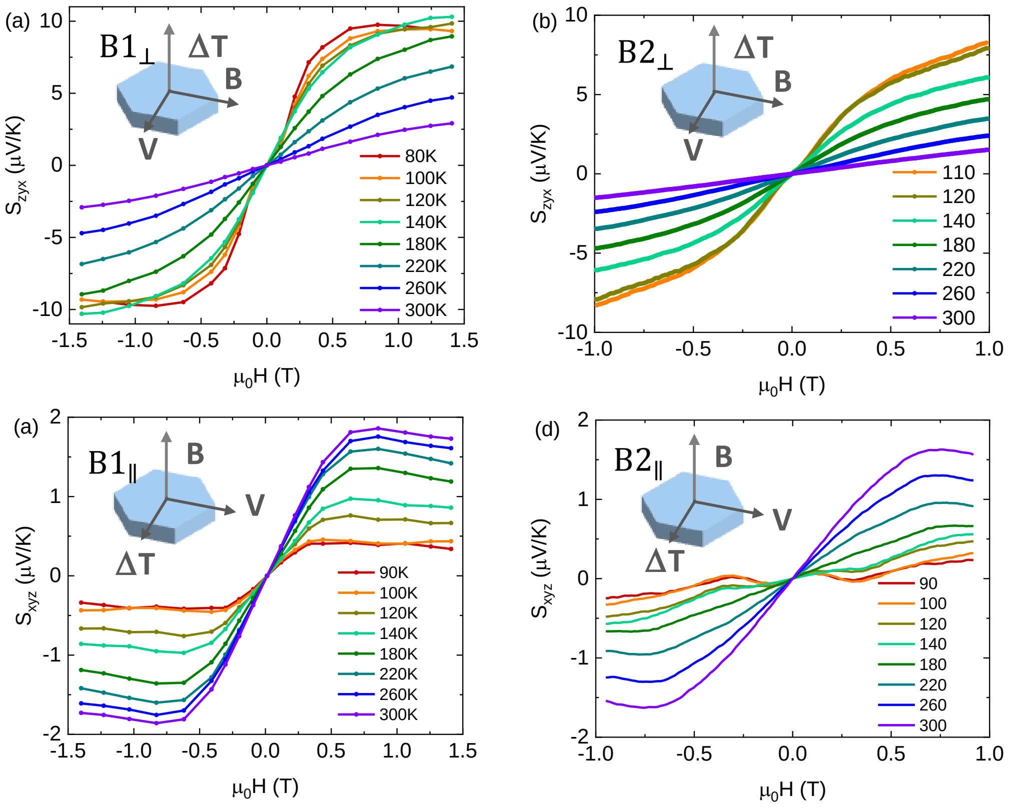

Figure S6: Complete dataset of the Nernst signal. (a)-(d) Nernst thermopower of four crystals corresponding to Figure 2 (a)-(d) in the main text. Clear slope change on the Nernst singles are observed after saturation magnetization, as a consequence of ordinary Nernst effect. Noting the saturation behavior is not observed on sample B2⊥, so that we are unable to determine the ordinary Nernst contribution.

7. Complete dataset of the Hall signals

In this part we present the Hall signals before the subtraction of the anomalous part. Fig. S8 (a) – (d) show the Hall resistivities after anti-symmetrization from the raw data. We observe a slightly different field dependence of the Hall effect compared to the Nernst effect. We attribute field dependence of the Hall resistivity to the complex magnetic structure during the spin reorientation. Since the ANE is dominated by the magnon contribution, it is less affected by the spin reorientation process.

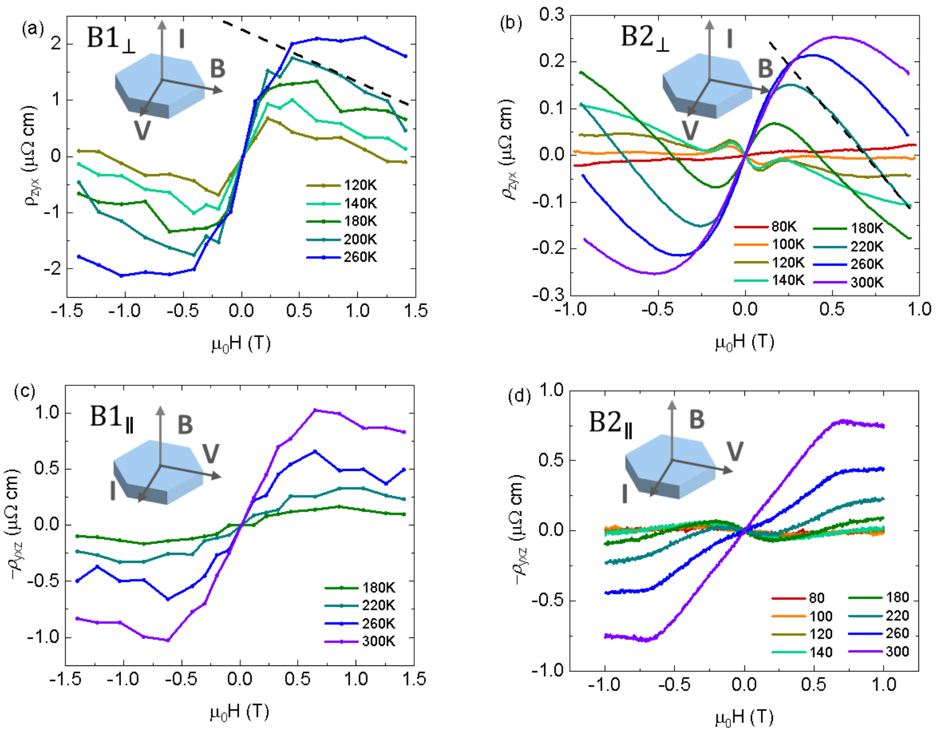

Figure S7: Complete dataset of the Hall signal. (a)-(d) Raw data of the Hall resistivities corresponding to Fig. 3(a)-(d) in the main text. Non-linear behaviors can be clearly seen in the Batch-2 samples, arising from the spin-reorientation. The black dash line schematically shows the ordinary Hall effect at higher field.

8. Temperature normalized ANE thermopower

To further prove the extrinsic magnonic contribution to the ANE signal, we plot the S_{zyx}/T as at various temperatures, which is then compared with the ANE thermopower. In the MnBi case, after dividing by T, the differences between the 80 K and other temperatures are more pronounced, which indicates that an extrinsic mechanism dominated the low temperature ANE signals. We believe this extrinsic contribution comes from magnons.

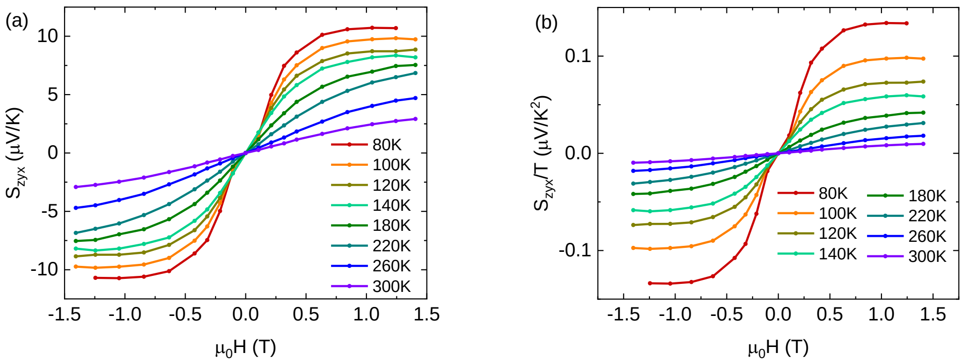

Figure S8: Pronounced magnon-drag induced ANE signal discovered via temperature normalization of the ANE curves. Comparison between the (a) ANE thermopower with (b) temperature-normalized ANE thermopower, which divides the ANE thermopower by the temperature. After normalization, the 80 K ANE signal becomes more significant compared to the higher temperature signals, indicative of low-temperature, extrinsic contributions, which is very likely to originate from magnons.
