# Supplementary material for: Large magnon-induced anomalous Nernst conductivity in single-crystal MnBi
Source: Joule. 2021 Nov 17;5(11):3057–67. doi: 10.1016/j.joule.2021.08.007 (PMC8604385; doi:10.1016/j.joule.2021.08.007)
Supplement: Document S2. Article plus supplemental information [file mmc2.pdf]

Joule

CellPress
OPEN ACCESS

Article

Large magnon-induced anomalous Nernst conductivity in single-crystal MnBi

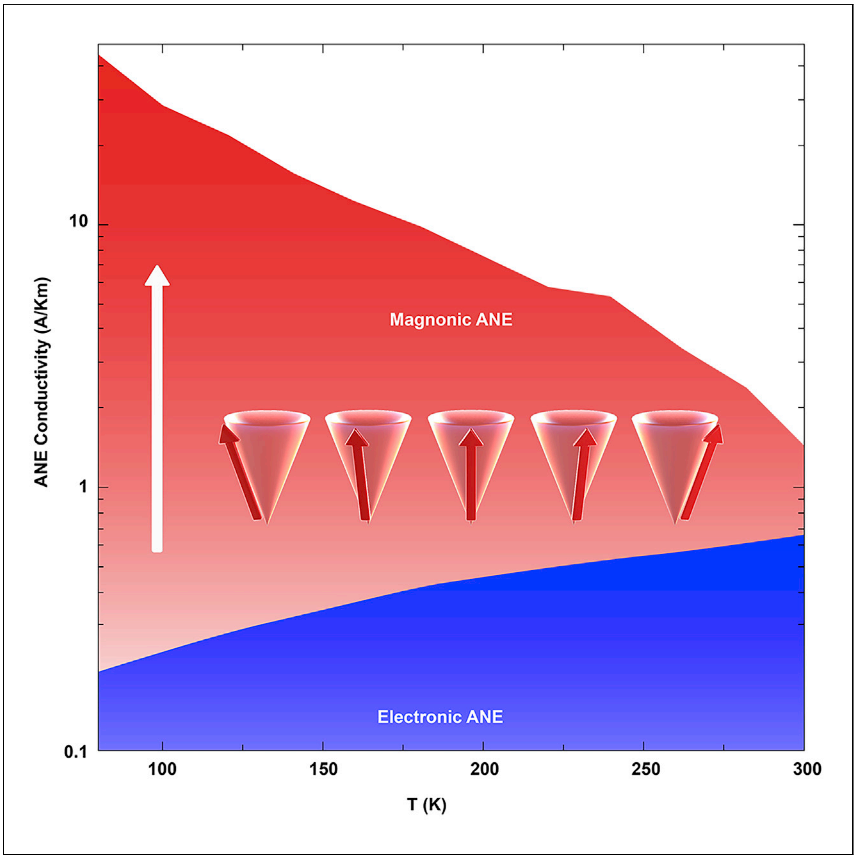

Bin He, Cüneyt Şahin, Stephen R. Boona, ..., Claudia Felser, Michael E. Flatté, Joseph P. Heremans

bin.he@cpfs.mpg.de

Highlights

MnBi shows a remarkable anomalous Nernst thermopower of 10 μV/K at 80 K

Anomalous Nernst conductivity reaches 40 A/Km, the highest value reported

Magnon-electron drag is likely the source of the large anomalous Nernst effect

Transverse thermoelectrics utilizing the anomalous Nernst effect (ANE) can be a novel approach to energy sustainability. We investigate the thermoelectric transport properties in ferromagnetic MnBi and observe one of the largest ANEs ever reported. We attribute this giant ANE to the coexistence of ferromagnetism and the heavy Bi atom. Our discovery proposes an alternative recipe to generate large ANE, which introduce a large spin-orbit coupling to ferromagnetic systems.

He et al., Joule 5, 3057–3067
November 17, 2021 © 2021 The Authors.
Published by Elsevier Inc.
https://doi.org/10.1016/j.joule.2021.08.007

Joule

CellPress OPEN ACCESS

Article

Large magnon-induced anomalous Nernst conductivity in single-crystal MnBi

Bin He,[1,2,9,*] Cüneyt Şahin,[3,4] Stephen R. Boona,[5] Brian C. Sales,[6] Yu Pan,[1] Claudia Felser,[1] Michael E. Flatté,[3,4] and Joseph P. Heremans[2,7,8]

SUMMARY

Thermoelectric modules are a promising approach to energy harvesting and efficient cooling. In addition to the longitudinal Seebeck effect, transverse devices utilizing the anomalous Nernst effect (ANE) have recently attracted interest. For high conversion efficiency, it is required that the material have a large ANE thermoelectric power and low electrical resistance, which lead to the conductivity of the ANE. ANE is usually explained in terms of intrinsic contributions from Berry curvature. Our observations suggest that extrinsic contributions also matter. Studying single-crystal manganese-bismuth (MnBi), we find a high ANE thermopower (~10 μV/K) under 0.6 T at 80 K, and a transverse thermoelectric conductivity of over 40 A/Km. With insight from theoretical calculations, we attribute this large ANE predominantly to a new advective magnon contribution arising from magnon-electron spin-angular momentum transfer. We propose that introducing a large spin-orbit coupling into ferromagnetic materials may enhance the ANE through the extrinsic contribution of magnons.

INTRODUCTION

With its ability to convert heat directly into electricity and vice versa, thermoelectricity plays a significant role in energy harvesting as well as static cooling applications.[1–4] Since over 90% of the energy humanity uses today comes from thermal processes, even a very slight improvement in efficiency translates into a large amount of energy saved, for example, by recovering the heat wasted in the exhaust of internal-combustion engines. To date, most thermoelectric research has focused on the longitudinal Seebeck effect, in which the temperature gradient is parallel to the voltage generated. In this case, the thermopiles have to be connected in series to generate a high voltage. This requires that electrical contacts be made at the hot side of each of the n- and p-type elements of each couple, a task that requires the development of a contact technology that minimizes thermal diffusion of the contact material into the thermoelectric. The need for individual contacts to each thermocouple elements and the fact that all these contacts are connected in series in the assembled module results in irreversible efficiency loss in contact resistance. Transverse thermoelectric devices, in which the voltage generated is perpendicular to the applied temperature gradient, can avoid these disadvantages. This configuration vastly simplifies the fabrication procedure by making it possible to apply the electrical contact only to a colder side of the thermoelectric material. It also reduces the thermal resistance, where a designated voltage can be generated by simply making the device longer or thicker. The low contact resistance losses of transverse devices compared with Peltier coolers

Context & scale

Thermoelectricity offers the prospect of generating electric power from heat and controlling temperatures in a directed manner. The oldest thermoelectric devices, dating to the 1800s, were based on Seebeck's observation of electric fields forming in the direction of a temperature gradient. More recent devices have been based on the transverse thermoelectric effect, in which the voltage and thermal gradients are perpendicular. In the anomalous Nernst effect, a magnetic field normal to a thermal gradient produces a voltage drop transverse to both. This Hall-like effect is poorly understood but has great potential for applications in thermoelectric energy harvesting and cooling. We demonstrate a startlingly large transverse thermoelectric response in MnBi crystals, which traditional approaches cannot fully explain. We believe that it is due to interactions between magnons and electrons. This mechanism may operate in other materials, allowing major advances in thermoelectrics.

 © 2021 The Authors. Published by Elsevier Inc.
This is an open access article under the CC BY license (http://creativecommons.org/licenses/by/4.0/).

is also an asset on cooling applications. With increasing demand for microdevice cooling, it is essential to achieve large transverse thermoelectric response and reveal the mechanisms to maximize the thermoelectric power output.

Transverse thermoelectrics utilizing the anomalous Nernst effect (ANE) have attracted much attention in recent years.[5–11] In ANE, the thermoelectric voltage, applied temperature gradient, and external field are perpendicular to each other, making it promising for transverse thermoelectric applications. ANE can be viewed as the thermal analog to the anomalous Hall effect (AHE) in magnetic materials. Like the AHE signals, the ANE signal reaches its largest value at the saturation magnetization. ANE has been observed in many magnetic materials, particularly ferromagnets.[5–8,11] With recent progress in understanding the topology of magnetic materials, it is believed that the net Berry curvature is the origin of intrinsic ANE.[5,6,10,12] A further approach to enhancing the ANE thermopower is to utilize extrinsic, e.g., magnon contributions.[13] One well-established magnon-mediated transport phenomenon is the spin Seebeck effect (SSE),[14–19] observed in heterostructure thin film devices. In the SSE, a spin current is thermally excited in a ferromagnetic insulator and injected into a detection layer (Pt) with large spin-orbit coupling (SOC), where a transverse voltage is detected via the inverse spin Hall effect (ISHE).[20,21] Inspired by the SSE, we expect a magnonic contribution to the total ANE thermopower when introducing large SOC into magnetic materials, since the SSE and ANE share the identical geometry.

To observe a magnon-induced ANE, a large SOC is always required. We decided to focus on magnets with bismuth (Bi), the heaviest stable element with the largest SOC.[22] However, Bi-based ferromagnets have always been rare: Bi has difficulty reacting with 3*d* metals. To date, MnBi is the only know binary ferromagnetic bismuthide with a high Curie temperature (~630 K),[23] making it potentially a high-temperature permanent magnet. Historically the transport properties of MnBi were long uninvestigated due to the lack of single crystals.[24,25] In 2014, McGuire et al. published a study of single-crystal MnBi that detailed their successful single-crystal growth using the flux method.[26] Combining large SOC and ferromagnetism, single-crystal MnBi is an ideal system to investigate the magnon-induced advective contributions to ANE.

Here, we present our thermomagnetic measurements and the observation of a large ANE signal on two batches of MnBi single crystals, batch-1 (B1) and batch-2 (B2). For each batch, we examine two pieces of crystals for both the in-plane (//) and cross-plane (⊥) properties. We find a large ANE that reaches 10 μV/K at 0.6 T and 80 K. Moreover, the anomalous Nernst conductivity reaches over 40 A/Km (higher than any reported value), as a result of low resistivity and large ANE thermopower. By carefully comparing the experimental data with the tight-binding calculations, we confirm that the intrinsic ANE mechanism is not sufficient to explain the observation. We posit that the large ANE may arise from an additional advective transport process induced by magnons: the thermally driven magnon current may spin-polarize the conduction electrons dynamically, resulting in an additional transverse voltage due to large SOC in MnBi.

RESULTS

A scenario of how magnon-electron interactions can contribute to a large ANE is schematically shown in Figure 1A. When a temperature gradient is applied to a ferromagnetic material, magnons are excited and can interact with electrons in

[1]Max Planck Institute for Chemical Physics of Solids, Dresden 01187, Germany

[2]Department of Mechanical and Aerospace Engineering, The Ohio State University, Columbus, OH 43210, USA

[3]Pritzker School of Molecular Engineering, University of Chicago, Chicago, IL 60637, USA

[4]Optical Science and Technology Center and Department of Physics and Astronomy, the University of Iowa, Iowa City, IA 52242, USA

[5]Center of Electron Microscopy and Analysis, The Ohio State University, Columbus, OH 43210, USA

[6]Materials Science and Technology Division, Oak Ridge National Laboratory, Oak Ridge, TN 37830, USA

[7]Department of Materials Science and Engineering, The Ohio State University, Columbus, OH 43210, USA

[8]Department of Physics, The Ohio State University, Columbus, OH 43210, USA

[9]Lead contact

*Correspondence: bin.he@cpfs.mpg.de

https://doi.org/10.1016/j.joule.2021.08.007

CellPress
OPEN ACCESS

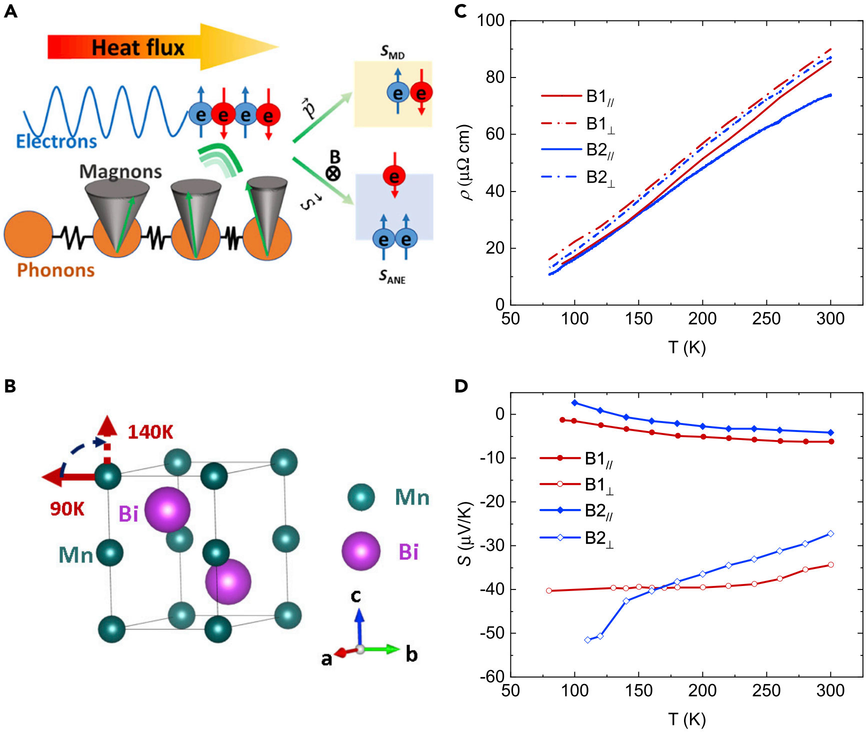

Figure 1. Overview of the experiment and longitudinal transport properties

(A) Schematic drawing of the magnon-induced advective transport. When a temperature gradient is applied to a ferromagnet, it is transferred from the phonon system to the magnon system. The magnons can then carry linear momentum ($\vec{p}$) and spin angular momentum ($\vec{S}$). Transfer of ($\vec{p}$) generates the magnon-drag thermopower S_{MD}. In a material with large SOC, transfer of $\vec{S}$ creates an out-of-equilibrium additional spin polarization of the conduction electrons that can generate a transverse thermoelectric voltage via the inverse spin Hall effect; this contributes to the ANE.

(B) Crystal structure of MnBi, with the preferential spin orientation labeled in red arrow. From 90 to 140 K, the spins reorient from *ab*-plane to *c* axis.

(C) Resistivity of four crystals $B1_{//} - B2_{\perp}$ measured from 80 to 300 K, with B1 and B2 stand for batch-1 and batch-2 crystals. The sample-to-sample variation in resistivity is possibly from different carrier concentration.

(D) Seebeck coefficient of four samples $B1_{//} - B2_{\perp}$. In-plane thermopowers have a positive temperature dependence, whereas the cross-plane thermopowers show a negative temperature dependence, which we speculate is related to the S_{MD}. Again, due to different carrier density, B1 samples have higher thermopower than B2 samples.

multiple ways further affecting the thermoelectric transport behavior. In the zero-field case, the magnons can transfer their linear momenta ($\vec{p}$) to the electrons and generate an advective contribution to the Seebeck coefficient, known as magnon-drag thermopower (S_{MD}).[27] In the presence of a magnetic field, magnon spin flux can transfer spin-angular momenta ($\vec{S}$) to the itinerant electrons during magnon-electron scattering in the bulk of the FM itself, thereby dynamically spin-polarizing the itinerant electrons beyond what is expected from the thermodynamic equilibrium band structure. This corresponds to spin pumping across an interface in the SSE, except that here the spin pumping occurs in the bulk during scattering processes. This electron polarization then can generate a transverse electric field by ISHE: $E_{ISHE} = D_{ISHE}(J_S \times \sigma)$, where D_{ISHE} stands for ISHE efficiency and is determined by the intensity of the SOC. Overall, this additional contribution to the ANE could be labeled a self-SSE term. Since Bi is known to exhibit very large SOC,[22] MnBi should have a large D_{ISHE}, giving rise to a potentially large extrinsic contribution to the ANE.

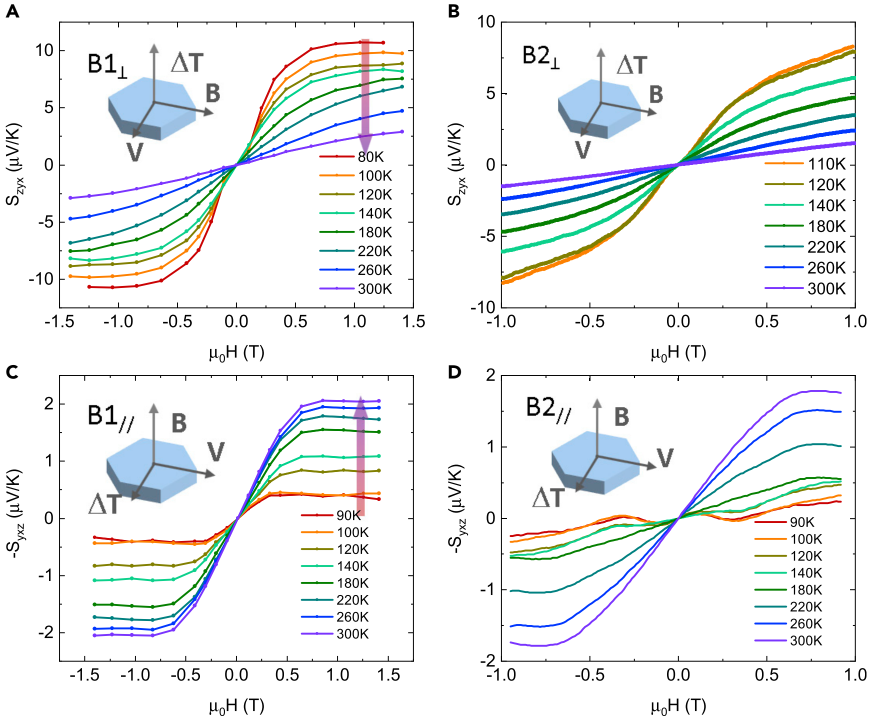

Figure 2. Field-dependent anomalous Nernst thermopower of MnBi along different directions

(A–D) Cross-plane ANE S_{zyx} on samples (A) B1$_\perp$ and (B) B2$_\perp$, with applied field parallel to *a* axis, and in-plane ANE S_{xyz} on samples (C) B1$_{//}$ and (D) B2$_{//}$, with applied field parallel to *c* axis. Measurements were taken down to the 90 K for B2$_{//}$, below which the magnet anisotropy teared apart the sample. On sample B2$_\perp$, due to the small size of the crystal (~ 1.2 mm), we were able to create measurable temperature gradient at 110 K. For S_{xyz}, the average temperature rise is ~ 7 K with the sink temperature at 110 K. At 100 K, the average temperature rise is over 10 K when a stable measurable temperature gradient is created. For this reason, the thermoelectric transport measurements are terminated at 110 K for B2$_\perp$. For the longer B1$_\perp$, we were able to measure down to 80 K without overheating the sample. The complete field-dependent Nernst thermopower is shown in Figure S6, from which a clear sign change of the ordinary Nernst signal is observed.

Figure 1B shows the crystal structure and the spin reorientation (SR) process of MnBi, with Mn as the smaller green sphere and Bi as the larger purple sphere. It crystalizes in hexagonal NiAs structure. The magnetic structure of MnBi is complicated because of its SR process.[26] Below 90 K, the spins are aligned in the *a*-*b* plane; above it, they start to rotate toward the *c* axis. By 140 K, SR is completed. Because it can change the normal modes of the magnons, SR has a significant influence on the ANE in MnBi

The resistivities of our four crystals increase with temperature, showing a metallic behavior (Figure 1C). A slight difference in resistivity has been observed from batch to batch, a result of different carrier concentrations induced by the different starting composition for the flux method (see section S1 for details). Such differences have also been observed in the Seebeck coefficients. Figure 1D shows the Seebeck coefficient of the four crystals. The in-plane Seebeck coefficients (B1$_{//}$ and B2$_{//}$) increase as temperature goes up, while the cross-plane thermopowers (B1$_\perp$ and B2$_\perp$) show the opposite trend. The latter behavior is unusual for a metal, and a tentative explanation is presented along with the Nernst discussion. The measurements on the two pairs of freshly prepared samples (B1$_{//}$ and B2$_{//}$) and (B1$_\perp$ and B2$_\perp$) indicate good sample-to-sample reproducibility. Meanwhile, thermopowers of B2 crystals are slightly lower than those of the B1, because of slightly higher carrier concentration.

CellPress
OPEN ACCESS

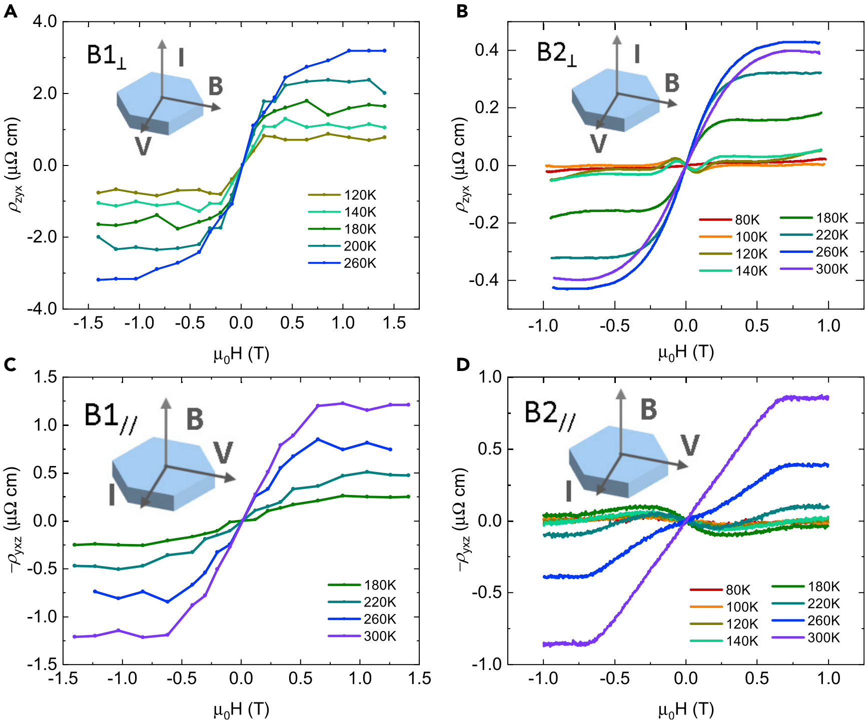

Figure 3. Anomalous hall effect in MnBi

(A–D) Cross-plane anomalous Hall resistivities of two cross-plane samples measured on (A) $B1_{\perp}$ and (B) $B2_{\perp}$, with the field applied along *a* axis, and in-plane AHE resistivities measured on samples (C) $B1_{//}$ and (D) $B2_{//}$, with applied field parallel to *c* axis. Nonlinear signals are detected on the field sweep measurement of B2 sample, possibly because of complex magnetic structure induced by spin reorientation and a weak first-order phase transition. We show the complete Hall curves in the supplemental information (Figure S7), from which the ordinary Hall signal is resolved.

Lastly, it should be mentioned that the samples are air sensitive; the thermoelectric transport properties, for instance—the Seebeck coefficient and ANE thermopower—degrade with repeated thermal cycling as described in the supplemental information (Figures S3 and S4).

Large ANE signals were observed in both batches of crystals. Figures 2A and 2B show the cross-plane ANE thermopower, S_{zyx}, measured on the samples $B1_{\perp}$ and $B2_{\perp}$. The largest S_{zyx} is observed on $B1_{\perp}$ at 80 K, reaching 10 μV/K at 0.6 T. Noting the complex magnetization process at low temperature, the magnetization curve is carefully analyzed, and the saturation field is confirmed to be 0.6 T at 80 K by the M–H curve (Figures S2A and S2B). Such a large ANE thermopower is higher than most reported values[5–9,11] and is only second to a recently reported compound, $UCo_{0.8}Ru_{0.2}Al$.[28] The S_{zyx} of $B2_{\perp}$ reaches ~7 μV/K at 110 K, comparable with that of $B1_{\perp}$ in the same temperature range. For $B1_{\perp}$, the S_{zyx} can reach saturation at 1 T from 80 to 140 K. When the SR process is completed, our crystals do not reach saturation magnetization at 1.4 T; thus no saturation in S_{zyx} is observed on $B1_{\perp}$ from 180 to 300 K. At room temperature, our measured ANE thermopower is 2.5 μV/K for $B1_{\perp}$ and 1.5 μV/K for $B2_{\perp}$. Figures 2C and 2D show the in-plane ANE thermopower S_{yxz}, measured on $B1_{//}$ and $B2_{//}$. For both samples, a clear saturation field of 0.8 T is observed above 140 K. The largest S_{yxz} values of both crystals are around 2 μV/K at 300 K. A nonlinear ANE signal is observed below 140 K on $B2_{//}$, which possibly originates from a complex magnetic structure induced by the SR process.

Since the origins of intrinsic AHE and ANE are often related to each other, we examined the AHE signals of all four crystals in addition to the ANE. Figures 3A and 3B show the cross-plane AHE resistivities ρ_{zyx} on $B1_{\perp}$ and $B2_{\perp}$ at various temperatures. Clear AHE signals have been observed on both samples. The AHE signal of $B1_{\perp}$ is larger than the signal of $B2_{\perp}$, which is possibly due to different carrier concentrations and scattering effects. Figures 3C and 3D show the in-plane AHE resistivities ρ_{yxz} on $B1_{//}$ and $B2_{//}$. Clear AHE signals are observed above 180 K on both samples, with a clear nonlinear ρ_{yxz} on $B2_{//}$ during the SR.

Currently, the net Berry curvature is considered to be the intrinsic mechanism of anomalous transverse transport properties, with skew scattering and side jump as the extrinsic mechanisms of AHE.[29] The net Berry curvature is considered to dominate the good metal region ($10^4 \sim 10^6$ S/cm), while the extrinsic skew scattering dominates the high conductivity region (>10^6 S/cm) and side jump dominates the bad metal region (<10^4 S/cm). Since the resistivity of MnBi falls in the good metal region, the Berry curvature should dominate the signals in AHE. Experimentally, we observed a nonlinear Hall behavior below 0.3 T in AHE resistivities of the B2 samples (Figures 3B and 3D), which is because of the complex magnetic structure arising from the SR. Additionally, a weak first-order phase transition[26] would also affect the magnetic structure. A detailed study of the nonlinear Hall effect is beyond the scope of this paper, but it can be achieved in the future.

In contrast with the AHE signals, the nonlinear ANE signals is only observed on the in-plane measurement (Figure 2D), but not on the cross-plane (Figure 2B). We believe that the nonlinear signals observed in both in-plane AHE (Figure 3D) and ANE (Figure 2D) share the same origin: the Berry curvature shifting during SR. However, for the cross-plane measurements, the nonlinearity is only found on AHE (Figure 3B), but not on ANE (Figure 2B), we therefore speculate that the net Berry curvature is not the dominant mechanism for the ANE. An extrinsic mechanism with a large transverse signal is participating in the ANE. Extrinsic contributions to Nernst effect can arise from interactions between charge carriers and quasiparticles, including magnon drag,[30] paramagnon drag,[31–33] and phonon drag.[34] Since magnons have been experimentally detected in MnBi single crystals,[35] we propose they are playing a significant role in thermomagnetic transport.

DISCUSSION

When the charge carriers are in thermodynamic equilibrium (i.e., in the absence of advective transport processes, such as drag), the ANE is related to the energy derivative of the AHE via the Mott relation in metals and degenerately doped semiconductors.[36] However, the Mott relation breaks down in the presence of drag contributions because it assumes an electron energy distribution that follows equilibrium Fermi-Dirac statistics.[36,37] Under drag conditions, the electron population, taken in isolation, is not at equilibrium. Since the Mott relation permits the calculation of an intrinsic contribution to the ANE that has the same origin as that of the AHE, we first derive the tensor elements of the experimental thermoelectric conductivity tensor $\overleftrightarrow{\boldsymbol{\alpha}}$ at various temperatures. This tensor $\overleftrightarrow{\boldsymbol{\alpha}}$ is related to the thermopower tensor $\overleftrightarrow{S}$ and the conductivity tensor $\overleftrightarrow{\boldsymbol{\sigma}}$, and in particular, the anomalous Nernst conductivity $\alpha_{xy} = S_{xx}\sigma_{yx} + S_{xy}\sigma_{xx}$.

Figure 4A shows the temperature dependent ANE thermopowers from 80 to 300 K. The in-plane ANE thermopower (S_{yxz}) of both batches increased with temperature, whereas the cross-plane ANE thermopower (S_{zyx}) decreased monotonically with

CellPress
OPEN ACCESS

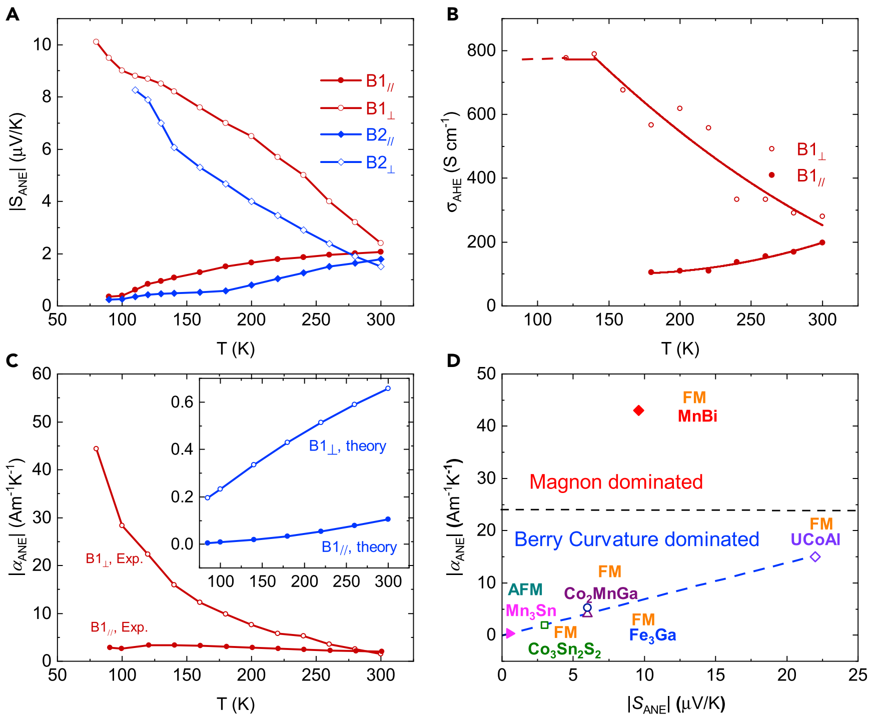

Figure 4. Giant transverse thermoelectric conductivity in MnBi single crystal

(A) Temperature dependence of anomalous Nernst thermopower. For in-plane direction, data are taken at 0.8 T. For cross-plane direction, data are taken at saturation field below 140 K, and 1 T above 140 K.

(B) Anomalous Hall conductivity calculated with measured longitudinal and transverse resistivities. Points are calculated σ_{AHE}; curves are a guide to the eye.

(C) Theoretical and experimental transverse thermoelectric linear response tensor element α_{yz} and α_{xy}, comparing with tight-binding calculated by the tight-binding Hamiltonian.

(D) Comparison of the experimental α_{yz} and S_{yz} with other magnetic materials. The giant α_{yz} is a result of low resistivity and large anomalous Nernst thermopower. The anomalous Nernst conductivity of MnBi is outstanding compared with other magnetic materials because of the magnon-mediated transport signature. The dashed lines are a guide to the eye.

temperature, from a maximum of 10 μV/K at 80 K to about 2 μV/K at room temperature. Figure 4B shows the anomalous Hall conductivities (AHCs) on $B1_{\perp}$ and $B1_{//}$. (Note that we are particularly interested in B1 because of the larger ANE signals.) The cross-plane AHC is approximately 800 S/cm below 140 K, while above 140 K it decreases with temperature because of non-saturation behavior. The in-plane AHC increases with temperature, reaching ~ 200 S/cm at 300 K. The AHCs in both directions fall in the intrinsic region of the AHE, which is consistent with the analysis on the longitudinal electrical conductivity.

With the acquired AHCs, we calculated the in-plane and cross-plane thermoelectric linear response tensor elements ($\alpha_{//}$ and $\alpha_{\perp}$), and compared them with tight-binding results for B1 in Figure 4C. Experimentally, $\alpha_{\perp}$ decreased with temperature, with the largest value of ~44 A/Km at 80 K. Such a large transverse thermoelectric conductivity is an order of magnitude higher than known ferromagnets and antiferromagnets.[5–8] In theory, both the intrinsic $\alpha_{//}$ and $\alpha_{\perp}$ should increase with temperature (shown in the embedded figure), and have the absolute values between 0 and 1 A/Km, much smaller than the experimental results. In addition, we examined the $\alpha_{\perp}$ with the Fermi energy shifting from −5 to 5 eV, however, the absolute value of $\alpha_{\perp}$ never exceeds 1 A/Km (Figure S5). Thus, the intrinsic contribution does not by itself explain the behavior of the $\alpha_{\perp}$

sufficiently; magnon-mediated transport can cause a much larger transverse thermoelectric response. In a magnon-mediated transport process, large ANE thermopower and longitudinal conductivity are able to coexist because magnons are Bosons, and they are not subjected to the Fermi-Dirac distribution. The large ANE is related to the magnon population and magnon-electron interaction, not to the electronic band structure. With a large ANE thermopower and a low resistivity, we achieve a giant α_{ANE}. The observed temperature dependence gives further evidence for this interpretation. At low temperature, the local spins are aligned in the *ab*-plane, and ΔT is applied along the *c* axis. The temperature gradient can excite the magnons and give rise to the self-SSE after the domains are aligned by an external field. In this case, self-SSE can be the origin of the giant Nernst thermopower in $B1_{\perp}$. Between 90 and 140 K, the local spins start to rotate from *ab*-plane to *c* axis, and the magnon dispersion relation also begin to change. The spin orientation reduces the total number of magnons excited by the temperature gradient along the *c* axis direction, so the self-SSE signal should decrease with temperature. We show the temperature normalized ANE thermopower in Figure S8, which is supportive to the proposal of low temperature magnon-drag effect.

This advective picture is also consistent with the fact that the longitudinal cross-plane thermopower decreases with increasing temperature (Figure 1C), assuming a large magnon-drag component. According to a previous study,[27] in a simple model the magnon drag thermopower and magnon drag ANE are related to each other by the formula $S_{ANE} = C\mu_0 H \frac{S_{md}}{\rho}$, where C is a material parameter depending on the effective mass and scattering mechanism, μ_0H is the applied field, S_{MD} is the magnon-drag thermopower and ρ is the resistivity. Experimentally, both longitudinal and transverse thermopowers decrease with temperature, which agrees qualitatively with the prediction of the formula above. Such a decreasing trend is indicative of the magnon-drag contribution to both longitudinal and transverse thermoelectric response. Lacking the detailed band parameters and scattering parameters, we are unable at this time to further quantify the value of C.

Finally, in Figure 4D we compare our experimental ANE thermopower S_{ANE} and transverse thermoelectric conductivity α_{ANE} with other magnetic materials with large ANE responses.[5–9,28] MnBi's S_{ANE} is about 25% higher than that of Fe_3Ga and Co_2MnGa, for both of which the intrinsic contributions are recognized as the origin of the large ANE signal. More importantly, the anomalous Nernst conductivity of MnBi is outstanding among all magnetic materials, reaching over 40 A/Km. Even compared with $UCo_{0.8}Ru_{0.2}Al$ with a larger ANE thermopower, our experimental α_{ANE} is still three times higher than that of $UCo_{0.8}Ru_{0.2}Al$. With the extrinsic magnon-drag contribution, MnBi can have the large ANE thermopower while maintaining a low longitudinal resistivity. Such a large α_{ANE} suggests that this magnon-drag-induced spin angular momentum transfer procedure is a highly effective approach to generating a large transverse thermoelectric response, which can be a new strategy for high-performance themoelectric applications.

In summary, MnBi, with a large SOC as well as strong ferromagnetism, is an ideal candidate for studying magnon-mediated transport phenomena, and therefore achieving large transverse thermoelectric response. A large ANE signal of 10 μV/K and a record anomalous Nernst conductivity of over 40 A/Km were observed in single-crystal MnBi at 80 K. This giant transverse thermoelectric response arises from an extrinsic magnon-electron interaction process. The magnon-electron spin angular momentum transfer process significantly enhances the ANE signal, equivalent to a self-SSE. Our study provides a new fundamental understanding of ANE, which can be quite large in ferromagnets with strong spin-orbit interaction. Such utilization of the magnon-electron

CellPress
OPEN ACCESS

interactions provides routes for the enhancement of ANE, and will doubtless find applications, as well as be generalized to other topics in the thermoelectrics field.

EXPERIMENTAL PROCEDURES

Single crystals of MnBi were grown at Oak Ridge National Lab (ORNL) using the method detailed in McGuire et al[26]; they are called Batch-1 (B1) in this paper. The crystals grown at Max Plank Institute for Chemical Physics of Solids (CPfS) were grown in the same method with a slightly higher starting Mn molar fraction (9%); they are called Batch-2 (B2). Optical image, Laue diffraction result and other details is reported in section S1, Figure S1, and Table S1. MnBi single crystals grow as hexagonal cylinders. We measured the transport properties in a modified Janis liquid nitrogen flow cryostat, as well as a quantum design physical properties measurement system with a breakout box. In both measurement systems, the set-ups were identical as described previously.[38] We report our result on $B1_{//}$ and $B2_{//}$ for the in-plane transport properties and $B1_{\perp}$ and $B2_{\perp}$ for the cross-plane properties. The temperature dependent magnetization is measured in a quantum design magnetic properties measurement system 3 from 80 to 300 K, up to 5 T.

We use the following notation for the transverse transport properties, which are denoted by indices *xyz*. Here, *x* is the direction of the applied thermodynamic flux (charge or heat flux), *y* is the direction of the measured voltage, and *z* is the field direction. Thus, S_{yxz} is the in-plane Nernst thermopower with the magnetic field along the z [0001] axis and measured on crystal $B1_{//}$ / $B2_{//}$, while S_{zyx} is the cross-plane Nernst thermopower with the field parallel to the *a* axis $[2\overline{1}\overline{1}0]$ in the hexagonal lattice, measured on crystals $B1_{\perp}$/$B2_{\perp}$. The sign convention in this experiment is opposite to the Gerlach sign conventions.

We constructed a tight-binding Hamiltonian using the parameters derived from the Density Function Theory.[39] MnBi has a point group of D_{6h} and a space group of *P*63, with a hexagonal crystal structure and 4 atoms per unit cell. The lattice constants were taken as $a = b = 4.285$ Å and $c = 6.113$ Å. The tight-binding Hamiltonian consisted of *p*-orbitals of Bi and *d*-orbitals of Mn. Magnetism was incorporated into the Hamiltonian through the Stoner formalism with parameters 4.5 and 0.2 eV for *d*- and *p*-orbitals, respectively. We also have added the spin-orbit Hamiltonian with spin-orbit couplings of 0.048 and 1.4 eV for Mn and Bi, respectively. The Berry curvature Ω_{ij}, which is an intrinsic property of the electronic band structure, was computed from this 16-band tight-binding Hamiltonian by:

$$\Omega_{ij}(nk) = Im\sum_{n\neq n'}\frac{<u_{nk}\left|\frac{\partial H}{\partial k_i}\right|u_{n'k}><u_{n'k}\left|\frac{\partial H}{\partial k_j}\right|u_{nk}>}{(\varepsilon_n-\varepsilon_{n'})^2}$$

For zero magnetization (or no external magnetic field) d-orbitals are located densely around 0 eV energy. As magnetization is increased with applied field, bands that predominantly consist of d-orbitals move away from the center to higher and lower energies. The energy-resolved AHC is calculated by integrating the Berry curvatures

$$\sigma_{ij} = -\frac{e^2}{\hbar}\int dk\sum_n\Omega_{ij}(nk)f_{nk}$$

where Ω_{ij} is the Berry curvature, and the summation is performed over all occupied bands in the first Brillouin zone. The intrinsic contribution to the anomalous Nernst conductivity (ANC) is related to the sum of the Berry curvatures and can be calculated by the well-established relation[40]:

$$\alpha_{ij} = -\frac{1}{e}\int d\varepsilon \frac{\partial f(\varepsilon)}{\partial \mu}\sigma_{ij}(\varepsilon)\frac{\varepsilon - \mu}{T}$$

where e is the electric charge, f is the Fermi-Dirac distribution, T is the temperature, σ denotes the energy-resolved intrinsic AHC, and μ is the chemical potential.

Resource availability

Lead contact

Further information and requests for resources and materials should be directed to and will be fulfilled by the lead contact, Bin He (bin.he@cpfs.mpg.de).

Materials availability

This study did not generate new unique materials.

Data and code availability

All data from this study are available from the lead contact upon reasonable request.

SUPPLEMENTAL INFORMATION

Supplemental information can be found online at https://doi.org/10.1016/j.joule.2021.08.007.

ACKNOWLEDGMENTS

B.H. and J.P.H. acknowledge support from the Center for Emerging Materials, an NSF MRSEC grant (DMR-2011876). B.H., Y.P., and C.F. acknowledge support from ERC TOPMAT, the European Union (grant no. 742068), and European Union's Horizon 2020 research and innovation program (grant no. 766566). C.Ş. and M.E.F. acknowledge support from the Center for Emergent Materials, an NSF MRSEC under award no. DMR-1420451. B.C.S. acknowledge support from the U.S. Department of Energy, Office of Science, Basic Energy Sciences, Materials Sciences and Engineering Division.

AUTHOR CONTRIBUTIONS

Conceptualization, B.H., S.R.B., and J.P.H.; experiments, B.H., S.R.B., B.C.S., and Y.P; calculations, C.Ş. and M.E.F.; analysis and discussion, B.H., C.Ş., S.R.B., Y.P., C.F., M.E.F., and J.P.H.; writing and revision, B.H., C.Ş., S.R.B., B.C.S., Y.P., C.F., M.E.F., and J.P.H.

DECLARATION OF INTERESTS

The authors declare no competing interests.

Received: April 7, 2021
Revised: June 14, 2021
Accepted: August 20, 2021
Published: September 14, 2021

REFERENCES

1. Goldsmid, H.J. (2010). Introduction to Thermoelectricity (Springer).

2. Bell, L.E. (2008). Cooling, heating, generating power, and recovering waste heat with thermoelectric systems. Science *321*, 1457–1461.

3. Snyder, G.J., and Toberer, E.S. (2008). Complex thermoelectric materials. Nat. Mater. *7*, 105–114.

4. Heremans, J.P., Dresselhaus, M.S., Bell, L.E., and Morelli, D.T. (2013). When thermoelectrics reached the nanoscale. Nat. Nanotechnol. *8*, 471–473.

5. Sakai, A., Minami, S., Koretsune, T., Chen, T., Higo, T., Wang, Y., Nomoto, T., Hirayama, M., Miwa, S., Nishio-Hamane, D., et al. (2020). Iron-based binary ferromagnets for transverse thermoelectric conversion. Nature *581*, 53–57.

6. Sakai, A., Mizuta, Y.P., Nugroho, A.A., Sihombing, R., Koretsune, T., Suzuki, M.-T., Takemore, N., Ishii, I., Nishio-Hamane, D., Arita, R., et al. (2018). Giant anomalous Nernst effect and quantum critical scaling in a ferromagnetic semimetal. Nature Phys *14*, 1119–1124.

7. Guin, S.N., Vir, P., Zhang, Y., Kumar, N., Watzman, S.J., Fu, C., Liu, E., Manna, K., Schnelle, W., Gooth, J., et al. (2019). Zero-field

CellPress
OPEN ACCESS

Nernst effect in a ferromagnetic Kagome-lattice Weyl-semimetal Co3Sn2S2. Adv. Mater. *31*, 186022.

8. Guin, S.N., Manna, K., Noky, J., Watzman, S.J., Fu, C., Kumar, N., Schnelle, W., Shekhar, C., Sun, Y., Gooth, J., and Felser, C. (2019). Anomalous Nernst effect beyond the magnetization scaling relation in the ferromagnetic Heusler compound CO2MnGa. NPG Asia Mater *11*, 16.
9. Ikhlas, M., Tomita, T., Koretsune, T., Suzuki, M.-T., Nishio-Hamane, D., Arita, R., Otani, Y., and Nakatsuji, S. (2017). Large anomalous Nernst effect at room temperature in a chiral antiferromagnet. Nature Phys *13*, 1085–1090.
10. Liang, T., Lin, J., Gibson, Q., Gao, T., Hirschberger, M., Liu, M., Cava, R.J., and Ong, N.P. (2017). Anomalous Nernst effect in the Dirac semimetal Cd3As2. Phys. Rev. Lett. *118*, 136601.
11. Ramos, R., Aguirre, M.H., Anadón, A., Blasco, J., Lucas, I., Uchida, K., Algarabel, P.A., Morellón, L., Saitoh, E., and Ibarra, M.R. (2014). Anomalous Nernst effect of Fe3O4 single crystal. Phys. Rev. B *90*, 054422.
12. Sykora, S., Caglieris, F., Wuttke, C., Büchner, B., and Hess, C. (2018). Anomalous Nernst effect in Weyl semimetals TaP and TaAs. Phys. Rev. B *98*, 201107.
13. Papaj, M., and Fu, L. (2021). Enhanced anomalous Nernst effect in disordered Dirac and Weyl materials. Phys. Rev. B *103*, 075424.
14. Uchida, K., Takahashi, S., Harii, K., Ieda, J., Koshibae, W., Ando, K., Maekawa, S., and Saitoh, E. (2008). Observation of the spin Seebeck effect. Nature *455*, 778–781.
15. Uchida, K., Xiao, J., Adachi, H., Ohe, J., Takahashi, S., Ieda, J., Ota, T., Kajiwara, Y., Umezawa, H., Kawai, H., et al. (2010). Spin Seebeck insulator. Nat. Mater. *9*, 894–897.
16. Jaworski, C.M., Myers, R.C., Johnston-Halperin, E., and Heremans, J.P. (2012). Giant spin Seebeck effect in a non-magnetic material. Nature *487*, 210–213.
17. Uchida, K., Adachi, H., An, T., Ota, T., Toda, M., Hillebrands, B., Maekawa, S., and Saitoh, E. (2011). Long-range spin Seebeck effect and acoustic spin pumping. Nat. Mater. *10*, 737–741.
18. Jaworski, C.M., Yang, J., Mack, S., Awschalom, D.D., Myers, R.C., and Heremans, J.P. (2011). Spin-Seebeck effect: a phonon driven spin distribution. Phys. Rev. Lett. *106*, 186601.
19. Jaworski, C.M., Yang, J., Mack, S., Awschalom, D.D., Heremans, J.P., and Myers, R.C. (2010). Observation of the spin-Seebeck effect in a ferromagnetic semiconductor. Nat. Mater. *9*, 898–903.
20. Bauer, G.E.W., Saitoh, E., and van Wees, B.J. (2012). Spin caloritronics. Nat. Mater. *11*, 391–399.
21. Boona, S.R., Myers, R.C., and Heremans, J.P. (2014). Spin caloritronics. Energy Environ. Sci. *7*, 885–910.
22. Heremans, J.P., Cava, R.J., and Samarth, N. (2017). Tetradymites as thermoelectrics and topological insulators. Nat. Rev. Mater. *2*, 17049.
23. Adams, E., Hubbard, W.M., and Syeles, A.M. (1952). A new permanent magnet from powdered manganese bismuthide. J. Appl. Phys. *23*, 1207–1211.
24. Poudyal, N., and Ping Liu, J.P. (2013). Advances in nanostructured permanent magnets research. J. Phys. D: Appl. Phys. *46*, 043001.
25. Williams, H.J., Sherwood, R.C., and Boothby, O.L. (1957). Magnetostriction and magnetic anisotropy of MnBi. J. Appl. Phys. *28*, 445–447.
26. McGuire, M.A., Cao, H., Chakoumakos, B.C., and Sales, B.C. (2014). Symmetry-lowering lattice distortion at the spin reorientation in MnBi single crystals. Phys. Rev. B *90*, 174425.
27. Watzman, S.J., Duine, R.A., Tserkovnyak, Y., Boona, S.R., Jin, H., Prakash, A., Zheng, Y., and Heremans, J.P. (2016). Magnon-drag thermopower and Nernst coefficient in Fe, Co, and Ni. Phys. Rev. B *94*, 14.
28. Asaba, T., Ivanov, V., Thomas, S.M., Savrasov, S.Y., Thompson, J.D., Bauer, E.D., and Ronning, F. (2021). Colossal anomalous Nernst effect in a correlated noncentrosymmetric kagome ferromagnet. Sci. Adv. *7*, eabf1467.
29. Nagaosa, N., Sinova, J., Onoda, S., MacDonald, A.H., and Ong, N.P. (2010). Anomalous Hall effect. Rev. Mod. Phys. *82*, 1539–1592.
30. Blatt, F.J., Flood, D.J., Rowe, V., Schroeder, P.A., and Cox, J.E. (1967). Magnon-drag thermopower in iron. Phys. Rev. Lett. *18*, 395–396.
31. Zheng, Y., Lu, T., Polash, M.M.H., Rasoulianboroujeni, M., Liu, N., Manley, M.E., Deng, Y., Sun, P.J., Chen, X.L., Hermann, R.P., and Zhao, H. (2019). Paramagnon drag in high thermoelectric figure of merit Li-doped MnTe. Sci. Adv. *5*, eaat9461. https://doi.org/10.1126/sciadv.aat9461.
32. Ahmed, F., Tsujii, N., and Mori, T. (2017). Thermoelectric properties of CuGa1−xMnxTe2: power factor enhancement by incorporation of magnetic ions. J. Mater. Chem. A *5*, 7545–7554.
33. Vaney, J.-B., Aminorroaya Yamini, S., Takaki, H., Kobayashi, K., Kobayashi, N., and Mori, T. (2019). Magnetism-mediated thermoelectric performance of the Cr-doped bismuth telluride tetradymite. Mater. Today Phys. *9*, 100090.
34. Ziman, J.M. (1960). Electrons and Phonons (Clarendon Press).
35. Williams, T.J., Taylor, A.E., Christianson, A.D., Hahn, S.E., Fishman, R.S., Parker, D.S., McGuire, M.A., Sales, B.C., and Lumsden, M.D. (2016). Extended magnetic exchange interactions in the high-temperature ferromagnet MnBi. Appl. Phys. Lett. *108*, 192403.
36. Cutler, M., and Mott, N.F. (1969). Observation of Anderson localization in an electron gas. Phys. Rev. *181*, 1336–1340.
37. Heremans, J.P. (2020). Thermal spin transport and spin in thermoelectrics. arXiv, arXiv:2001.06366.
38. Heremans, J.P., Thrush, C.M., and Morelli, D.T. (2004). Thermopower enhancement in lead telluride nanostructures. Phys. Rev. B *70*, 115334.
39. Shanavas, K.V., Parker, D., and Singh, D.J. (2014). Theoretical study on the role of dynamics on the unusual magnetic properties in MnBi. Sci. Rep. *4*, 7222.
40. Xiao, D., Yao, Y., Fang, Z., and Niu, Q. (2006). Berry-phase effect in anomalous thermoelectric transport. Phys. Rev. Lett. *97*, 026603.

Joule, Volume *5*

Supplemental information

Large magnon-induced anomalous

Nernst conductivity in single-crystal MnBi

Bin He, Cüneyt Şahin, Stephen R. Boona, Brian C. Sales, Yu Pan, Claudia Felser, Michael E. Flatté, and Joseph P. Heremans

Supplemental Experimental Procedures

1 Single crystal characterization

High Quality single crystals are essential to study the thermoelectric transport properties of materials. In this study, the single crystal information of Batch 1 can be found from previous publication (Ref. 26 in the main text). In this part we show the crystal information of Batch-2 grown at the Max Planck Institute. Fig. S1(a) shows the optical image of MnBi single crystal from Batch-2, with a hexagon shape. Fig. S1(b) shows the Laue diffraction taken on the crystal. According to the Mn-Bi binary phase diagram, MnBi single crystals can be grown via flux method with a starting Mn mole fraction less than 10%. As described in the main text, Batch 1 and 2 were grown with 6% and 9% Mn respectively. By analyzing the ordinary Hall effect, they have carrier concentrations of ~ $8 \times 10^{20}/cm^3$ and $1.4 \times 10^{21}/cm^3$. We suspect the crystals are with different defect structures, which give rise to the different carrier concentrations. The defect structure is affected by the Mn chemical potential in the crystal growth process. To our knowledge, we believe a lower starting Mn composition is beneficial for low carrier concentration and larger thermoelectric/thermomagnetic response.

(a) (b)

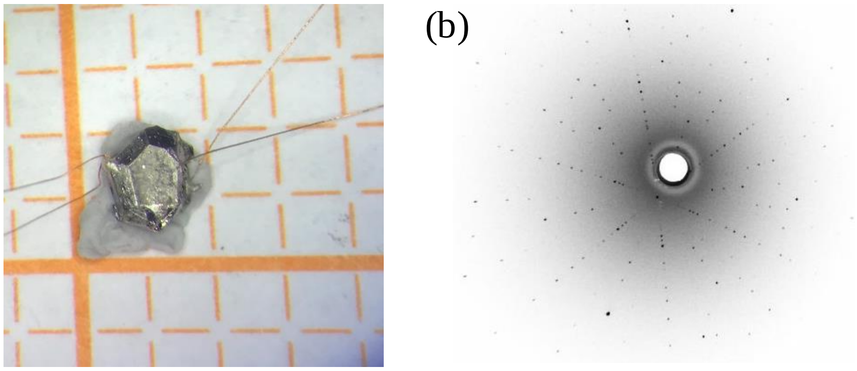

Figure S1: Characterization of single crystal MnBi. (a) Optical image and (b) Laue diffraction pattern on MnBi single crystal from Batch-2, indicative of high crystal quality

	Starting composition	Carrier density	Maximum S_{ANE}	Maximum α_{ANE}
Batch-1	6% Mn+94% Bi	$8 \times 10^{20}/cm^3$	10 μV/K	44A/Km
Batch-2	9% Mn+91%Bi	$1.4 \times 10^{21}/cm^3$	7 μV/K	~28A/Km

Table S1: Comparison between the crystals Batch-1 and Batch-2. With a higher starting Mn concentration, the carrier concentration of Batch-2 is higher than that of Batch-1, resulting in a smaller magnon-drag induced ANE thermopower and ANE conductivity.

Since batch 2 has a higher carrier concentration than batch 1, which then results in smaller magnon-drag transport signals. In the magnon-drag picture for longitudinal thermopower, the magnon-drag thermopower can be expressed as $S_{md} = \frac{2}{3}\frac{C_m}{ne}\frac{1}{1+\frac{\tau_{em}}{\tau_m}}$, where C_m is the magnon heat capacity, n is the carrier concentration, e is elemental charge, τ_{em} is the electron magnon scattering time and τ_m is the magnon mean free time. Generally, the magnon-drag thermopower is inversely proportional to the carrier concentration. The magnon-drag ANE may not have such simple relation. But it can be inferred that the magnon-drag ANE should be negatively related to the carrier concentration, which explains the sample to sample variation.

2 Magnetic Properties of MnBi

Fig. S2 (a) and (b) show the magnetization vs. field (M-H) at various temperatures when the applied field is parallel to *c*-axis and *ab*-plane, respectively. Our measurements mainly focus on the SR temperature range of 80 K and above, consistent with our transport measurement. Above 90 K, when the external field is applied parallel to *c*-axis, the spins can be easily aligned parallel to field, with the saturation field increasing upon cooling. The opposite trend has been observed when the field is applied in *ab*-plane. The crystal reaches its saturation magnetization at 80 K, with a small saturation field of 0.6 T. When the temperature is higher than 90 K, the saturation field scales with temperature, with the *c*-axis becoming the easy axis above 140 K. The saturation field is greater than 2 T at 180 K, which is beyond the max field we applied to our crystals. We also notice that, by applying high field, the crystals break into pieces after taken out from the MPMS. Thus, we do not apply field higher than 1.5 T for our transport measurement.

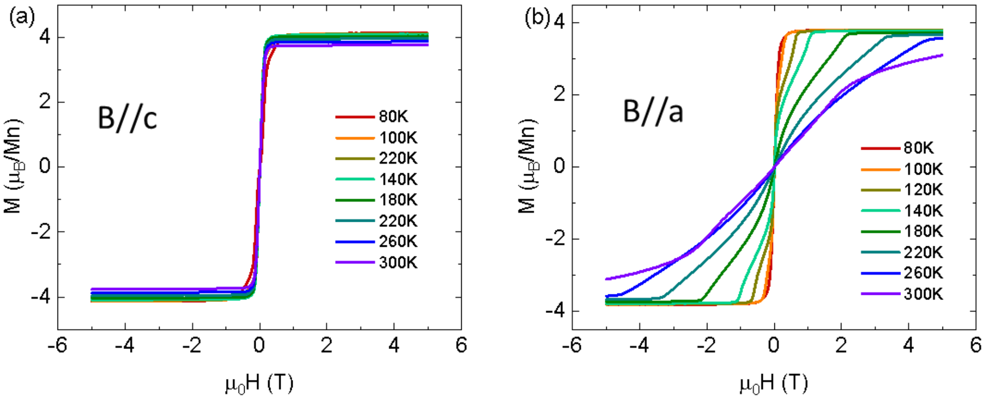

Figure S2: M-H results of MnBi single crystals. (a) M-H curve with external field along [0001]. (b) M-H curves with external field along $[2\bar{1}\bar{1}0]$. Data are taken on two small crystals from Batch-2. A clear spin-reorientation process is observed from M-H measurement.

3.Sample oxidation

The reproducibility of the transport data is established in the main text on freshly-grown samples. However, due to sample oxidation problems, it must be noted that the thermopower data change with the thermal cycling of the samples, and the Nernst data do so too although to a lesser extent. Compared to other manganese pnictides, MnBi single crystals have limited air stability because the electronegativity of Bi is small compared to other anions. We observed that after each thermal cycling, the surface of the MnBi crystals turned slightly yellow, indicative of the formation of bismuth oxide or manganese oxides. Thus, it is highly important to guarantee that the sample is mounted and measured in an air-free atmosphere to prevent sample oxidation and the accompanied weakening of the anomalous Nernst effect (ANE).

Here, we show the thermoelectric transport properties of sample $B1_{\perp}$ over three thermal cycles. We measured the sample multiple times for reproducibility; thus, we were able to observe a clear trend that both the ANE and thermopower decrease, which can come from oxidation of the sample. When MnBi crystals are oxidized, both the Mn and Bi are able to grab oxygen from the air, forming bismuth oxide Bi_2O_3, and complex manganese oxides (like MnO_2). This oxidation is creating defects and damaging sample quality. Oxidation of the crystal introduces a secondary phase and changes the carrier concentration of the sample, which can significantly affect the transport properties of the single crystal. Fig. S3(a) shows the anomalous Nernst thermopower of sample $B1_{\perp}$ at 100 K. The pristine sample had even larger anomalous Nernst thermopower in the first measurement. As we thermally cycled the sample in the cryostat, the anomalous Nernst thermopower reduced to 10 μV/K for the second thermal cycle. After we remounted the sample for the PPMS measurement, the anomalous Nernst thermopower further reduced to about 4 μV/K. As discussed above, this reduction in the ANE comes from the oxidation of the sample. The induced oxide impurities significantly scatter the electrons and reduce the mean free path of them. As the magnon electron interaction is a dynamical process, the extra scattering brings the system back to equilibrium, leading to a smaller anomalous Nernst thermopower. Fig. S3(b) shows the thermopower of sample $B1_{\perp}$ over three cycles. We observed a decrease in thermopower as we conducted the measurements, which is consistent with the vanishing ANE. The increasing density of defects deflects the magnon

electron interaction, which further reduces the magnon-drag thermopower. For the third run, the sample shows a metallic behavior, resulting from the disappearance of magnon-drag contribution.

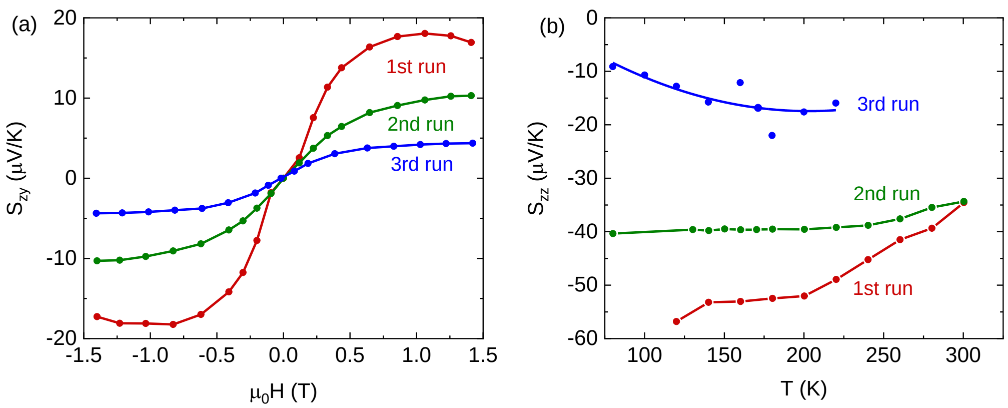

Figure S3: Thermal cycling results of thermoelectric transport coefficients. (a) Nernst thermopower of sample B1⊥ at 100 K after each thermal cycling. The anomalous Nernst thermopower decreases monotonically over thermal cycles. (b) Seebeck coefficient of B1⊥ after thermocycling. The absolute value of the Seebeck coefficient and anomalous Nernst coefficient decrease as the sample is oxidized and more defects are generated, possibly due to the vanishing of magnon-drag contribution.

4. Correlation between thermal and thermomagnetic transport properties

To further illustrate the possible contributions of magnons to the transport properties, we present the two samples with distinct thermal transport properties. We name our samples as B1⊥ and B1⊥*, where B1⊥ is the same sample as in the main text and B1⊥* is a sample which is unintentionally exposed to air for some time and thus got oxidized. As described in part S3, oxidation with damage the sample quality and reduce the ANE signal. Fig. S4(a) and S4(b) compare the ANE signals of the two samples. At 80 K, the ANE of B1⊥* is only 4 μV/K while the ANE of B1⊥ reaches 10 μV/K. As the temperature goes up, the ANE of B1⊥* increases with temperature as expected for intrinsic contribution, while the ANE of B1⊥ is almost constant over the spin reorientation process. At 160 K, the ANE of the two samples are almost identical to each other. The further decrease of B1⊥ is mainly due to non-saturation magnetization. However, since MnBi has strong magnetic anisotropy, we are unable to measured higher field results, as B1⊥* was teared apart by the applied field at 180 K. Moreover, the thermopower is also reduced as shown in Fig. S4(c). After being oxidized, the cross-plane thermopower shows a classic metallic signature, which is

increasing with temperature, with the absolute value much smaller than the pristine sample. Both of these two phenomena can be explained as the absence of magnon contribution. When the sample got oxidized, the magnon induced transport are suppressed, due to the higher defect density and smaller magnon and electron mean free path. At last, Fig. S4(d) shows the thermal conductivities of two samples B1⊥ and B1⊥*. After oxidation, the thermal conductivity is significantly reduced at low temperature, due to the much smaller contribution of magnon thermal conductivity.

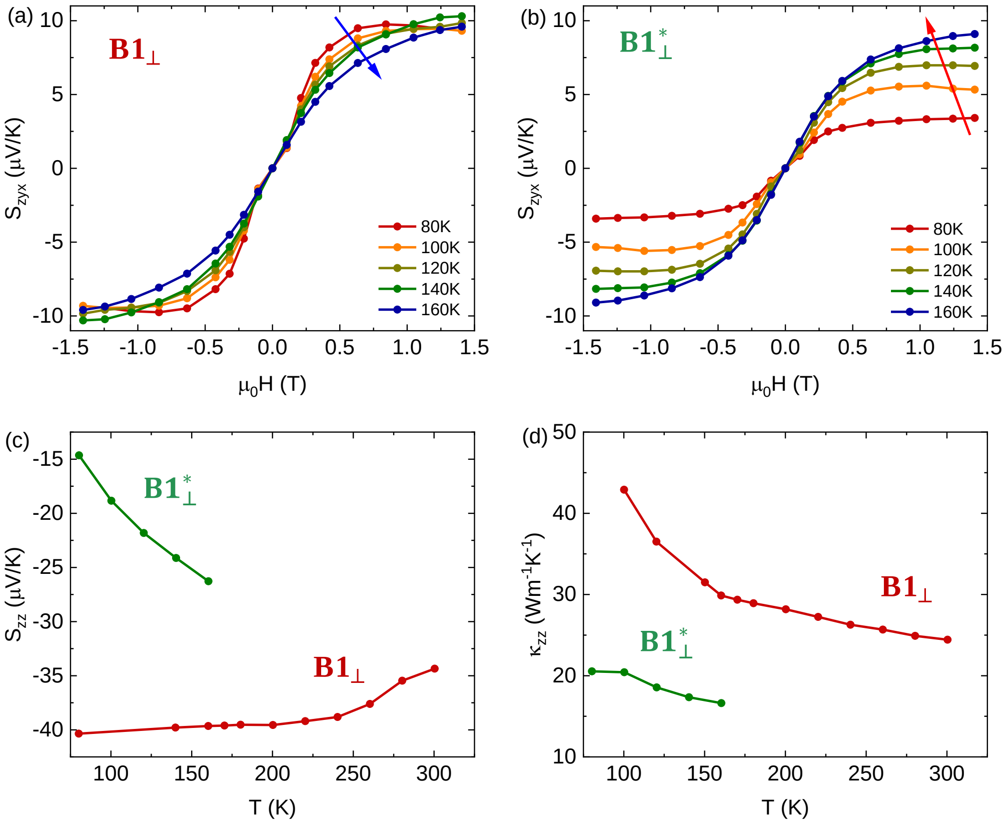

Figure S4: Relation between the large ANE thermopower and longitudinal Seebeck coefficient. (a) and (b) Nernst thermopower of sample B1⊥ and B1⊥* with opposite temperature dependence due to the unintentional oxidation of sample B1⊥*. (c) and (d) Seebeck coefficient and thermal conductivity comparison of samples B1⊥ and B1⊥*. We clearly see the large Nernst signal is accompanied with a larger Seebeck coefficient and higher thermal conductivity.

5. Tight-binding calculation with a variation of Fermi energy

In the tight -binding calculation, it is essential to locate the position of the Fermi energy, in order to calculate the transverse thermoelectric conductivity α_{zyx} and α_{xyz} . We

first calculate the cross-plane anomalous Hall conductivity (AHC) as a function of Fermi energy, shown in Fig. S4(a). By comparing the experimental result (800 S/cm) to the theoretical value, we locate our Fermi energy at the position of 0 eV. With this Fermi level position, the temperature dependent α_{zyx} and α_{xyz} and then calculated and shown in the main text Fig. 4(c). Furthermore, to eliminate the unintentional error from any slight variation of the Fermi energy, we calculate the energy dependent α_{zyx} from -5 eV to 5 eV. The maximum α_{zyx} is always below 1A/Km, which can not explain the experimental value.

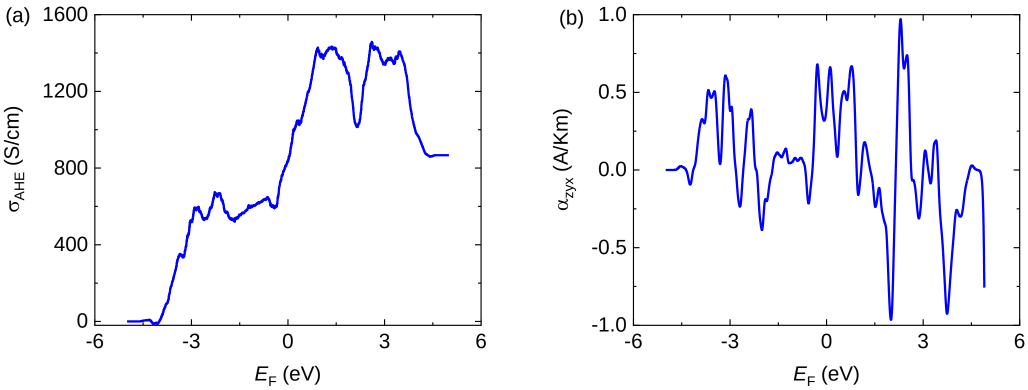

Figure S5: Energy dependent anomalous Hall and Nernst conductivities. (a) Cross-plane anomalous Hall conductivity (AHC) as a function of Fermi energy. Particularly when E_F=0 eV, the AHE is ~ 800 S/cm, which agrees with the experimental results. The Fermi energy is located at 0 eV for the transverse thermoelectric conductivity calculation. (b) Cross-plane transverse thermoelectric response element α in the tight-binding calculation, with the variation of the Fermi energy from -5 eV to 5 eV. In such a wide range of calculation, the calculated α is between -1 A/Km to 1 A/Km, indicative of the stability of the intrinsic contribution of the transverse thermoelectric response. Meanwhile, we conclude that, our giant transverse thermoelectric response cannot be explained by simply adjusting the position of the Fermi energy.

6. Complete dataset of the Nernst signals

In this part we present the Nernst signals before the subtraction of the anomalous part. Fig. S6 (a) – (d) show the total Nernst thermopower after anti-symmetrization from the raw data. We observe a clear slope change after saturation magnetization, as a result of ordinary Nernst effect (ONE). Meanwhile, as temperature goes up, we find a sign change of the ONE part. As the ONE is related to the scattering mechanism, there should be a change in the scattering mechanism before and after the spin reorientation effect.

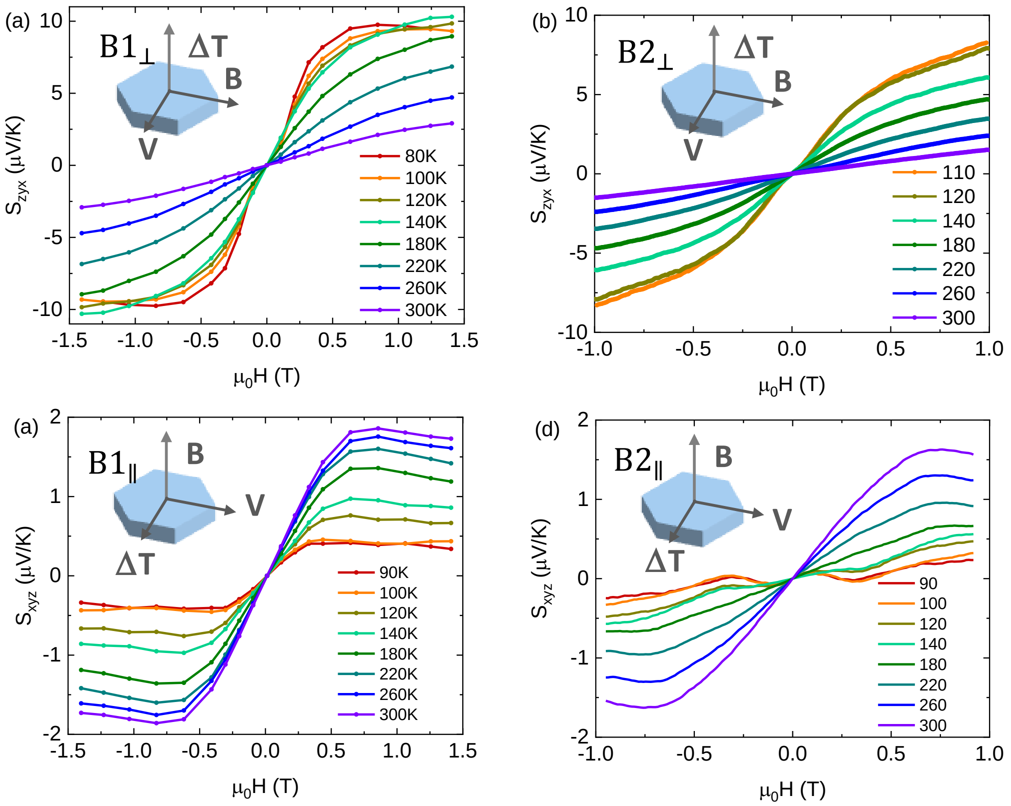

Figure S6: Complete dataset of the Nernst signal. (a)-(d) Nernst thermopower of four crystals corresponding to Figure 2 (a)-(d) in the main text. Clear slope change on the Nernst singles are observed after saturation magnetization, as a consequence of ordinary Nernst effect. Noting the saturation behavior is not observed on sample B2⊥, so that we are unable to determine the ordinary Nernst contribution.

7. Complete dataset of the Hall signals

In this part we present the Hall signals before the subtraction of the anomalous part. Fig. S8 (a) – (d) show the Hall resistivities after anti-symmetrization from the raw data. We observe a slightly different field dependence of the Hall effect compared to the Nernst effect. We attribute field dependence of the Hall resistivity to the complex magnetic structure during the spin reorientation. Since the ANE is dominated by the magnon contribution, it is less affected by the spin reorientation process.

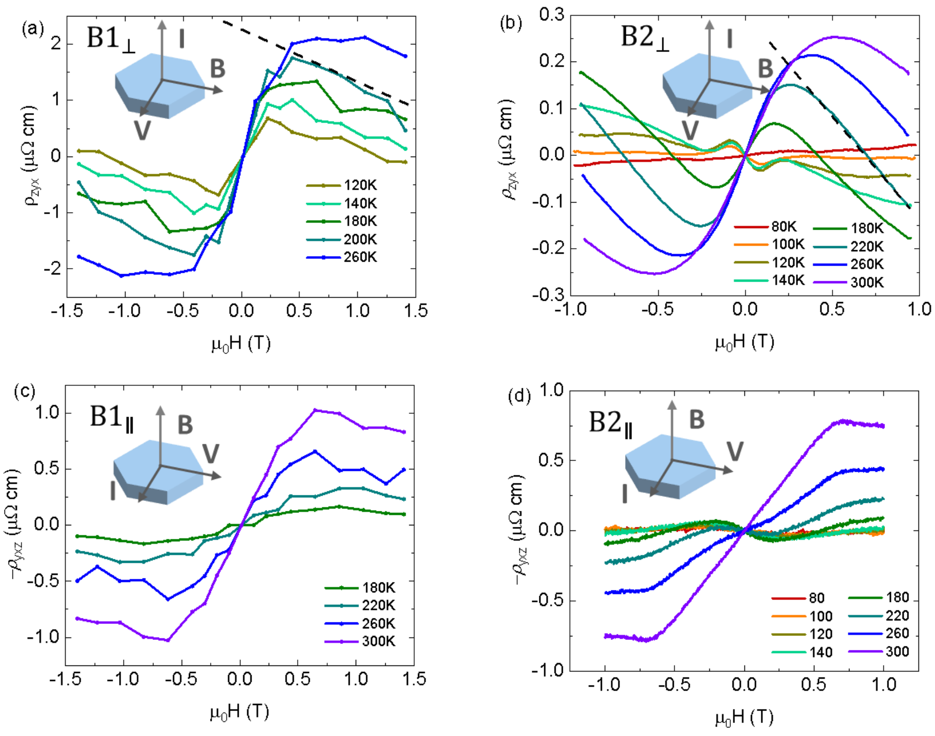

Figure S7: Complete dataset of the Hall signal. (a)-(d) Raw data of the Hall resistivities corresponding to Fig. 3(a)-(d) in the main text. Non-linear behaviors can be clearly seen in the Batch-2 samples, arising from the spin-reorientation. The black dash line schematically shows the ordinary Hall effect at higher field.

8. Temperature normalized ANE thermopower

To further prove the extrinsic magnonic contribution to the ANE signal, we plot the S_{zyx}/T as at various temperatures, which is then compared with the ANE thermopower. In the MnBi case, after dividing by T, the differences between the 80 K and other temperatures are more pronounced, which indicates that an extrinsic mechanism dominated the low temperature ANE signals. We believe this extrinsic contribution comes from magnons.

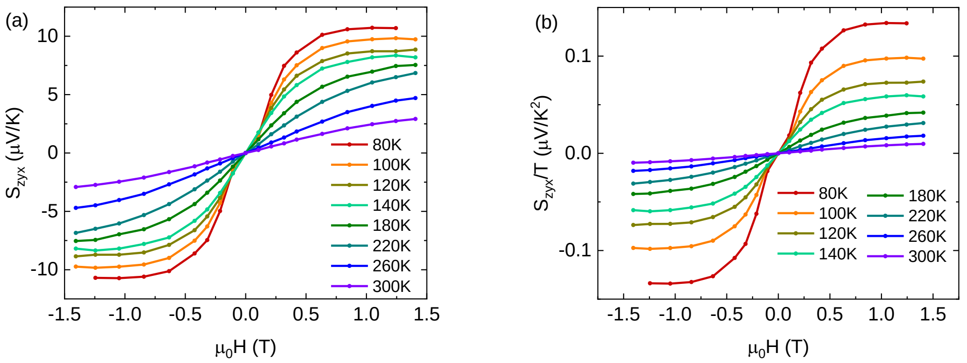

Figure S8: Pronounced magnon-drag induced ANE signal discovered via temperature normalization of the ANE curves. Comparison between the (a) ANE thermopower with (b) temperature-normalized ANE thermopower, which divides the ANE thermopower by the temperature. After normalization, the 80 K ANE signal becomes more significant compared to the higher temperature signals, indicative of low-temperature, extrinsic contributions, which is very likely to originate from magnons.
